# Interactions between the R2R3-MYB Transcription Factor, *At*MYB61, and Target DNA Binding Sites

**DOI:** 10.1371/journal.pone.0065132

**Published:** 2013-05-31

**Authors:** Michael B. Prouse, Malcolm M. Campbell

**Affiliations:** 1 Centre for the Analysis of Genome Evolution & Function, Department of Cell & Systems Biology, University of Toronto, Toronto, Ontario, Canada; 2 Department of Biological Sciences, University of Toronto Scarborough, Toronto, Ontario, Canada; Cankiri Karatekin University, Turkey

## Abstract

Despite the prominent roles played by R2R3-MYB transcription factors in the regulation of plant gene expression, little is known about the details of how these proteins interact with their DNA targets. For example, while *Arabidopsis thaliana* R2R3-MYB protein *At*MYB61 is known to alter transcript abundance of a specific set of target genes, little is known about the specific DNA sequences to which *At*MYB61 binds. To address this gap in knowledge, DNA sequences bound by *At*MYB61 were identified using cyclic amplification and selection of targets (CASTing). The DNA targets identified using this approach corresponded to AC elements, sequences enriched in adenosine and cytosine nucleotides. The preferred target sequence that bound with the greatest affinity to *At*MYB61 recombinant protein was ACCTAC, the AC-I element. Mutational analyses based on the AC-I element showed that ACC nucleotides in the AC-I element served as the core recognition motif, critical for *At*MYB61 binding. Molecular modelling predicted interactions between *At*MYB61 amino acid residues and corresponding nucleotides in the DNA targets. The affinity between *At*MYB61 and specific target DNA sequences did not correlate with *At*MYB61-driven transcriptional activation with each of the target sequences. CASTing-selected motifs were found in the regulatory regions of genes previously shown to be regulated by *At*MYB61. Taken together, these findings are consistent with the hypothesis that *At*MYB61 regulates transcription from specific *cis*-acting AC elements *in vivo*. The results shed light on the specifics of DNA binding by an important family of plant-specific transcriptional regulators.

## Introduction

Much of plant growth and development is shaped by sequence-specific transcription factors, proteins that act in response to external and internal cues to modulate gene expression. The MYB family is the largest family of plant sequence-specific transcription factors, with greater than 100 family members in individual plant species [1], [2], [3], [4], [5], [6]. MYB transcription factors are recognised by the presence of the MYB domain, which comprises characteristic helix-helix-loop-helix repeats of approximately 50 amino acids. The MYB domain binds DNA in a sequence-specific manner and is highly conserved in yeast, vertebrates, and plants [7]. The MYB domain is normally found near the amino terminus of the protein, and generally contains either 1, 2, or 3 of the 50 amino-acid MYB repeat. R2R3-MYB proteins have two such repeats, and comprise the largest sub-family of the MYB family. Moreover, R2R3-MYB proteins are plant specific, regulating facets of plant growth, development and metabolism [3], [5], [8], [9], [10], [11], [12], [13], [14], [15].

While members of the R2R3-MYB family are being characterised in increasing numbers, these investigations largely focus on the involvement of a particular MYB in the manifestation of a specific plant phenotype. That is, most of these analyses do not extend to a more detailed examination of MYB function at the molecular level. Nevertheless, some general themes with respect to R2R3-MYB function at the molecular level are emerging [16]. For example, many R2R3-MYB transcription factors bind to DNA motifs that are enriched in adenosine (A) and cytosine (C) residues [8], [17], where guanine (G) residues are either absent or depleted [16], [18]. These motifs have been variously referred to as AC elements, H boxes, or PAL boxes [18], [19], [20], [21], [22], [23], [24], [25], [26], [27]. Some R2R3-MYB proteins function as transcriptional activators at these sites [15], [17], while others function as transcriptional repressors [28]. AC elements are relatively short, comprising 5 or 6 nucleotides, where 3 residues form a relatively invariant core [29], [30], [31]. R2R3-MYB proteins bind to AC elements in a manner that relies on specific amino acid residues in the R2R3-MYB domain [29], [30], [31], [32], [33]. To date, the details of such interactions have been relatively scant, aside from their putative involvement in the regulation of plant-specific gene expression.

*At*MYB61, a member of the *Arabidopsis thaliana* R2R3-MYB family of transcription factors, illustrates the involvement of R2R3-MYB family members in the regulation of plant-specific processes. *At*MYB61 is a pleiotropic regulator of three major facets of the plant transpiration system: xylem cell differentiation; lateral root outgrowth; and, stomatal aperture [13], [34]. *At*MYB61 modifies gene expression in response to diurnal cues so as to appropriately modify the aperture of stomata [13], the pore-like structures on leaf surfaces that enable gas exchange. Thus, *At*MYB61 plays a role in modifying the capacity to take up carbon dioxide for photosynthesis, while limiting the loss of water from the plant body. *At*MYB61 also alters gene expression in response to sugars, resulting in modification of plant architecture and cell wall structure [14], [35], [36]. As is the case for most R2R3-MYB transcription factors, the precise mechanisms that enable *At*MYB61 to bring about important changes in plant function are unknown. Furthermore, although *At*MYB61 has been shown to bind to certain consensus motifs [34], the preferential binding of *At*MYB61 has not yet been determined quantitatively.

Given that R2R3-MYB proteins are involved in a rich variety of plant-specific processes [2], it would be desirable to have a more detailed understanding of R2R3-MYB and DNA motif interactions. The work described herein focuses on the interplay between *At*MYB61 and its DNA target sequences. Cyclic amplification and selection of targets (CASTing), which enables identification of a transcription factor’s DNA-binding sites from a pool of random oligonucleotides, was used to identify target DNA-binding sites for *At*MYB61 [37]. The sequences identified served as a useful foundation to examine mechanisms responsible for *At*MYB61 sequence-specific binding, and to hypotheses about the roles these may play in shaping *At*MYB61 function *in vivo.*

## Materials and Methods

### Ethics Statement

Antibody generation was carried out in strict accordance with the Province of Ontario’s Animals for Research Act, and the requirements of the federal Canadian Council on Animal Care. The protocol was approved at the University of Toronto, which involved full committee review by the Local Animal Care Committee (LACC), followed by approval by the University of Toronto Office of Research Ethics, the University Veterinarian, and finally the University of Toronto Animal Care Committee (UACC) (Permit Number: 20007080, approved 14/01/08). All efforts were made to minimise suffering.

### Expression of Recombinant Protein in Bacteria

Recombinant *At*MYB61 protein was produced in *E. coli* using the coding sequence cloned in frame into the *Nde*I and *Bam*HI sites of the pET15b vector (Novagen). Recombinant *At*MYB61 protein was produced, extracted and affinity purified as described previously for pine MYB proteins [17].

### Antibody Production and Western Blot Analysis

Anti-*AtMYB61* polyclonal antibodies were produced against the recombinant fusion protein in rabbits as described previously [38]. Affinity-purified recombinant antigen was gel-purified on a 10% SDS-PAGE gel and shipped in phosphate buffered saline to University of Toronto BioScience Support Laboratories for antibody production. In brief, 2 rabbits were each injected a total of 4 times with 300 µg of antigen per injection over a 6 week period. Production bleeds were performed after nitrocellulose dot blot assays indicated acceptable titre.

For western blot analysis, total soluble protein extracts were separated by SDS-PAGE and transferred to Bio-Rad Laboratories Nitrocellulose Trans-Blot Transfer Medium (0.45 µm) by electrophoretic transfer (BioRad, Mississauga, ON, Canada). Chemiluminescent western blot analysis was performed on the filters with Invitrogen’s Western Breeze Chemiluminescent kit as described by the manufacturer (Invitrogen, Burlington, ON, Canada). Primary antibody dilutions were done at a final dilution of 1/20000.

### Cyclic Amplification and Selection of Targets (CASTing)

The CASTing assay was completed according to Wright *et al* [37]. CASTing was completed by incubating 15 µg of double stranded random olionucleotides (27 mers) flanked in between two constant priming sequences with the *At*MYB61 full length recombinant protein. This complex was added to a Protein G Dynabead (Invitrogen, Burlington, ON, Canada) plus post-injection *At*MYB61 antibody complex, causing the complex to immunoprecipitate. The immunoprecipitated complex was then washed 3 times, resuspended in 100 µL PCR buffer, boiled and then PCR amplified for 30 cycles with 15 pmol of forward and reverse primers. 10 µl of the amplified selected targets were kept for analysis and 90 µL were used to continue with the next cycle. This cycle was repeated four more times to select for *At*MYB61 consensus DNA target sequences. The selected targets were then cloned into Invitrogen’s pCR4 TOPO vector and sequenced (Invitrogen, Burlington, ON, Canada).

### MEME (Multiple Em for Motif Elicitation)

MEME was conducted as described previously [39]. The MEME filter criteria was set for a min/max motif width of 6, any number of repetitions of a single motif distributed among the sequences, and no restrictions on the number of motifs identified. This allowed for the identification of all over-represented hexamer sequences in the recovered CASTing-enriched oligonucleotides. Moreover, it allowed for the identification of repeats of over-represented hexamer sequences in a given CASTing-enriched oligonucleotide.

### Nitrocellulose Filter-binding Assay

The nitrocellulose filter-binding assay was conducted as described by Hall and Kranz [40]. The CASTing targets that were over-represented were ordered from Invitrogen and PCR amplified (Invitrogen, Burlington, ON, Canada). These PCR products were Qiagen nucleotide purified according to the Qiagen manufacturer (Qiagen, Toronto, ON, Canada). The cleaned up PCR products were then radioactively labelled with P-32 via primer extension and further Qiagen nucleotide purified according to the Qiagen manufacturer (Qiagen, Toronto, ON, Canada). The CPM levels were measured via a liquid scintillation counter to measure the incorporation of P-32 into the probe. The radioactively labelled probes were combined in a binding reaction with recombinant *At*MYB61 protein and passed through BioRad nitrocellulose filters (0.2 µm) (BioRad, Mississauga, ON, Canada). The relative binding of recombinant *At*MYB61 protein to the CASTing motifs and mutated AC-I sequences were recorded. The dissociation constants (K_d_) of the CASTing targets to *At*MYB61 were determined by GRAFIT program which linearised the nonlinear regression via scatchard plots to calculate the point at which half of the ligand was bound to *At*MYB61.

### Electrophoretic Mobility-Shift Assay (EMSA)

Recombinant *At*MYB61 protein was produced, extracted and affinity purified as described previously for pine MYB proteins [17]. EMSA conditions were exactly as described previously [8], [17] but using recombinant *At*MYB61 protein in place of pine MYB protein.

### Molecular Modelling

The tertiary structure of *At*MYB61 was predicted using the tool PHYRE [41]; www.sbg.bio.ic.ac.uk/phyre/html/index.html). PHYRE proposed that the resolved structure that shared the most homology to *At*MYB61 was the animal c-MYB DNA-binding domain, which was resolved previously with its DNA consensus motif (AACNG) by heteronuclear multidimensional NMR [31]. This solution structure was used to predict a 3D protein model of *At*MYB61 with an E-value of 3.8e-13 and an estimated precision of 100%. The two protein sequences were 44% alike using amino acid sequence alignment. The PBD (protein data bank) file recovered from the PHYRE analysis (PBD ID = c1msfC) was used to superimpose the predicted *At*MYB61 crystal structure with the c-MYB crystal structure using DaliLite [42]. The c-MYB protein was resolved along with its DNA binding sequence allowing one to predict the binding domain of *At*MYB61 using homology. The PBD files for the AC-I and NBS nucleotide motifs were created from the http://structure.usc.edu/make-na/server.html server. Using Pymol [43] the two structures were modelled and superimposed [44]. Polar interactions were determined using Pymol.

### Transcriptional Activation Assay

Transcriptional activation assays using yeast were as described previously [17], but substituting the *At*MYB61 coding sequence in place of pine MYB sequences. Transcriptional activation assays were conducted with three biologically independent replicates per condition.

## Results and Discussion

### *At*MYB61 Bound a Discrete Subset of DNA Target Sequences

**Figure 1:**
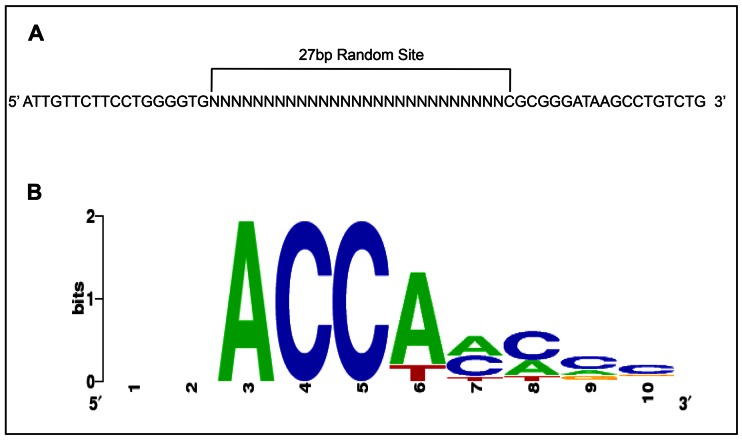
Cylic amplification and selection of targets (CASTing) recovered a suite of hexamer target sequences that bound to *At*MYB61. (A) 27 bp random sequences flanked by two primer sites (63 bp in total) were used in the CASTing assay. (B) Sequence logo of CASTing targets discovered by MEME. The ACC motif was conserved among all target sequences. Two nucleotides upstream and downstream of the over-represented hexamer target sequences were included to analyse if the over-represented motifs could be extended beyond a hexameric sequence.

To generate an antibody of adequate specificity for the cyclic amplification and selection of targets (CASTing) assay, antibodies were raised against a non-conserved region in the *At*MYB61 C-terminus (Figure S1). CASTing was initiated with a pool of 63-base-pair double-stranded oligonucleotides, where each oligonucleotide consisted of a segment of 27 random nucleotides flanked by designed sequences for PCR priming. A 15 µg (2.21×10^14^ DNA molecules) pool of “randomers” was incubated with *At*MYB61 full-length recombinant protein (Figure 1A). Assuming the average protein-binding site is a hexamer, the 27-bp degenerate core of each double-stranded oligomer contained 21 possible positions. Therefore, in the initial round of CASTing, 21×10^14^ unique sites were available for binding, when using 15 µg of randomers.

Five CASTing cycles were undertaken to enrich the pool of oligonucleotides in DNA binding-sites bound by *At*MYB61. The enriched oligonucleotides were cloned into pCR4 TOPO (Invitrogen, Burlington, ON, Canada) and sequenced. Following enrichment, 89 CASTing-derived oligonucleotides were sequenced. Sequences were subjected to analysis to discover over-represented motifs using MEME (Multiple Em for Motif Elicitation) [39] (Table 1, Table 2, Figure 1B). MEME filtering criteria identified sequences with a min/max motif width of 6, any number of repetitions of a single motif distributed among the sequences, and no restrictions on the number of motifs identified. Following MEME analysis, all CASTing-enriched sequences contained over-represented motifs characterised by an abundance of adenosine and cytosine residues. These over-represented motifs had a conserved set of ACC nucleotides present at the beginning of the motifs, suggesting that these nucleotides may be essential for recognition and binding (Table 1, Table 2, Figure 1B). These motifs correspond to canonical AC elements, also known as H-boxes or PAL-boxes (Table 1, Table 2, Figure 1B). Notably, a subset of CASTing-enriched oligonucleotides had multiple AC elements present in individual target sequences (Table 1). That said, a CASTing assay is a method to identify novel DNA-binding targets of a transcription factor of interest, and further characterisations are required to determine preferred targets.

**Table 1:** Alignment of *At*MYB61 binding sites obtained from CASTing Assay.

MEME (Multiple EM for Motif Elicitation) identified 7 over-represented hexamer motifs.			
Group		*At*MYB61 Site	
ACCACC			
1	ACCCCAGAGTCCC	ACCACC	CGACCCCC
2	ACCCAAACACCACGCCCTAG	ACCACC	C
3	GCTAAACGTTCATTCCCCT	ACCACC	CC
4	A	ACCACC	TCAACAAACCCCGGCCGCCC
5	ACCAC	ACCACC	ACCCACCCCCCCCCCC
6	G	ACCACC	CTCCAACCTATACCGGCCCC
7	CCAAACTCGACCGTTCCCGC	ACCACC	C
8	GCACCCC	ACCACC	ACCATACCTACCCC
9	ACCCGATCAGGCCCTCC	ACCACC	CCCC
10	CCACACCCCACCCCGAACG	ACCACC	GC
11	ACCAACGGACTAGCTCCCAC	ACCACC	C
12	C	ACCACC	CCACCATACAATCCCTAGGC
13	ACCAC	ACCACC	ACCCCACCCTAGGACC
14		ACCACC	ACTACCCGGACCCGGCCCCCC
15	ACACGAGATAACGACCCG	ACCACC	CCC
ACCTAC			
16	GACACAAGACAC	ACCTAC	ACCCCCCCC
17	GCAGCCC	ACCTAC	ACTCCCGCTCCCCC
18	GCACCCCACCACCACCAT	ACCTAC	CCC
19	ACCCCCCCTAATTG	ACCTAC	GGCAGGC
20	CAG	ACCTAC	CCCCGCCCCCAACCCGCC
21	CACCCACCGTCCAACG	ACCTAC	ACCCC
22	GCGCACCCCACCCCCC	ACCTAC	GGCCC
ACCACA			
23		ACCACA	ATGCAGCCGTACTTCGACCCC
24		ACCACA	CCACCACCCACCCCCCCCCCC
25	A	ACCACA	TCAACAAACCCCGGCCGCCC
26	CAACCCCTCCA	ACCACA	CCTCCCCGCC
27	CC	ACCACA	CTCTGCATTCTTGACCGCC
ACCATA			
28	GGGTAATGTC	ACCATA	GCCCCCCCCCC
29	GCACCCCACCACC	ACCATA	CCTACCCC
30	CA	ACCATA	CACAACGCCCCGACCCCCC
31	CACCACCCC	ACCATA	CAATCCCTAGGC
32	CAGGCACCCCCAACCCCCC	ACCATA	CC
ACCAAT			
33	AAAGGGTATACACAGGT	ACCAAT	GGCC
34	AACCTTAGGG	ACCAAT	CAATAAGGGAC
35		ACCAAT	GAAGAGACCCCTAACCATTAC
36	ATGTGTAG	ACCAAT	GGCATAATCTGCA
37	GTCGAGTCG	ACCAAT	GCAGCACGCAGC
ACCAAC			
38	CAG	ACCAAC	CTCATACCCCCCCCTGCC
39	CC	ACCAAC	CCTCCCTCCCAATGCCCGC
40		ACCAAC	GGACTAGCTCCCACACCACCC
41	AACATGCTGTGCAACCAA	ACCAAC	ACC
ACCAAA			
42		ACCAAA	AGATCAACCCCCCCCCGTACC
43	AACATGCTGTGCA	ACCAAA	CCAACGCC
44	ACACATAAACAGCA	ACCAAA	CCAGCCC
45	AACATGCTGTGCA	ACCAAA	CCAACACC

MEME (Multiple EM for Motif Elicitation) identified 7 over-represented hexamer motifs.

**Table 2:** *At*MYB61 consensus sequence was derived from a comparison 89 sequences recovered from 5 cycles of CASTing.

	−2	−1	A	C	C	W	H	H	+1	+2
**G**	3	11	–	–	–	–	–	–	9	7
**A**	10	8	45	–	–	38	20	14	10	4
**T**	2	3	–	–	–	7	5	5	2	4
**C**	20	17	–	45	45	–	20	26	24	27
**Total**			45	45	45	45	45	45		

The composition of each base at each position of the hexameric sequence is provided. −/+ indicate the bases 5′ or 3′ of hexameric consensus sequence. The bases 5′ or 3′ of sequences does not add up to 45 in certain circumstances because primer sites were negated from the analysis. W corresponds to A/T, H corresponds to A/T/C, – corresponds to a zero value.

AC elements, also known as PAL boxes or H-boxes, play key roles in regulating transcription for a variety of genes, particularly those encoding enzymes implicated in phenylpropanoid metabolism [18], [19], [20], [21], [22], [23], [24], [25], [26], [27]. R2R3-MYB proteins are known to bind AC elements and activate transcription from these motifs in yeast and *in planta* [16]. For example, pine (*Pinus taeda*) MYB1 [15] and MYB4 [17] and eucalyptus (*Eucalyptus grandis*) MYB2 [45], were all able to bind to AC elements present in the promoters of lignin biosynthetic genes. Similarly, pine (*Pinus taeda*) MYB1 and MYB4 bound AC elements present in the gene regulatory sequences of a pine gene encoding glutamate synthetase (*GS1b*) [8]. R2R3-MYB binding to AC elements is predicted to play a role in dictating xylem-localised expression of the aforementioned genes [8], [15], [17], [45]. Given the xylem-localised expression of *At*MYB61 [34], it is likely that it functions in an equivalent manner to drive AC-element-mediated expression in *Arabidopsis thaliana*.

### *At*MYB61 Bound to DNA Target Sequences with Varying Degrees of Affinity

**Figure 2:**
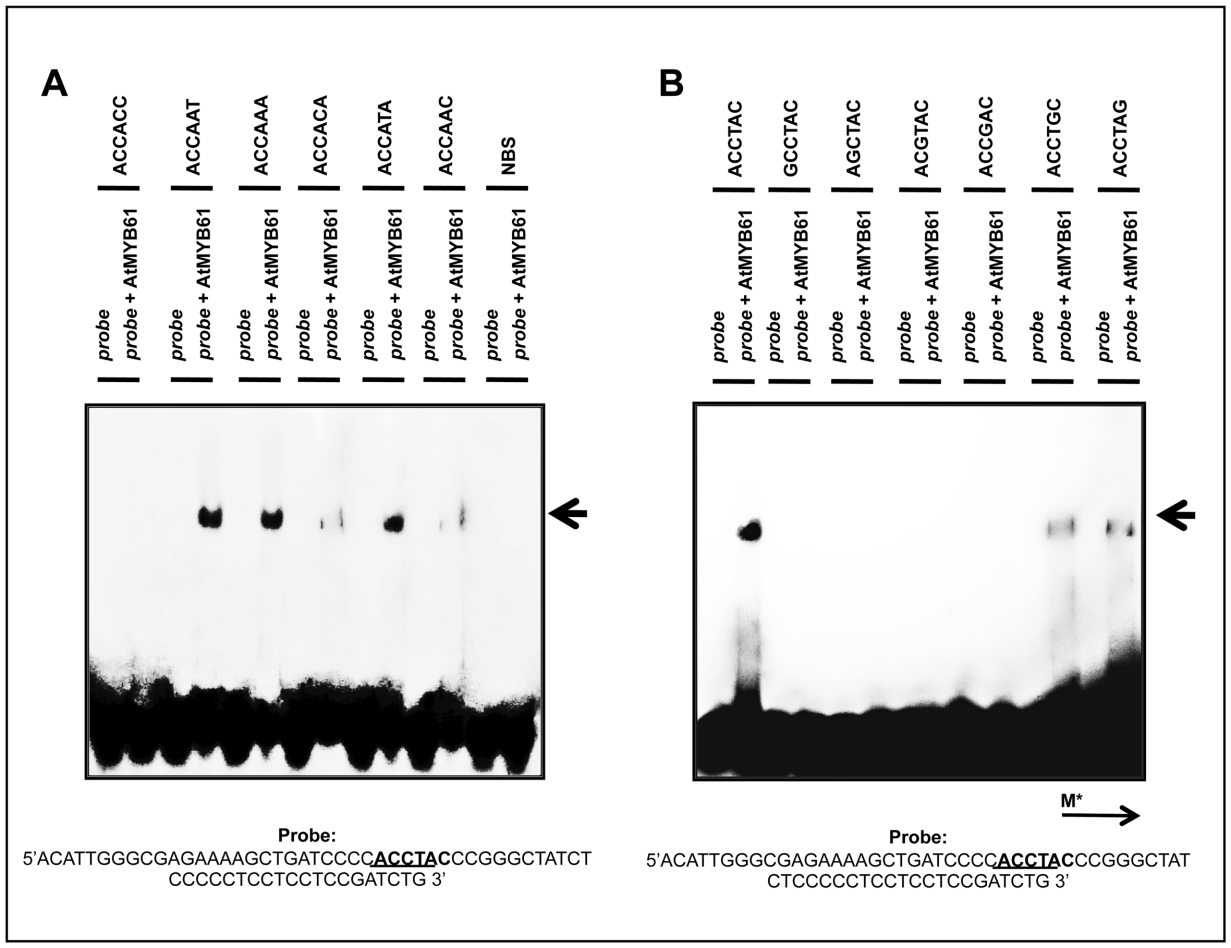
Relative binding affinities of *At*MYB61 to CASTing targets and to mutated ACCTAC motif determined by nitrocellulose filter-binding assays are confirmed by electrophoretic mobility-shift assays (EMSAs). (A) EMSA of recombinant *At*MYB61 protein binding to 6 labelled CASTing target sequences. The protein concentration used was 5×10–08M. Protein concentrations were conducted at 5×10–08M because this was the protein concentration at which targets had not all reached their binding max as determined by nitrocellulose filter-binding assay, allowing one to observe differential binding. (B) EMSA validating relative binding affinities of *At*MYB61 to mutated ACCTAC motif. The protein concentration used was 5×10–08M. Mutations were conducted by substituting a single guanine nucleotide along the AC1 element. Black arrow indicates gel shift by the probe. Non-binding site (NBS) is a sequence that does not bind *At*MYB61, acting as a negative control. Probes were engineered for the EMSA reaction by inserting the hexamer CASTing sequence or mutated AC1 element sequence into the underlined area.

The relative binding affinities of recombinant *At*MYB61 protein to the CASTing-derived sequences were determined (Table S1). Dissociation constants for each CASTing target were calculated by GRAFIT software program by using Scatchard plots (Table 3). The CASTing target that bound with the highest affinity (9.12E^−09 ^M) was ACCTAC (AC-I) (Table 3). Since the AC-I motif was the preferred target of *At*MYB61, a mutational assay was conducted on this motif to examine which nucleotides were essential for binding (Table 4). A guanine nucleotide was substituted one nucleotide at a time and shifted along the motif. A nitrocellulose filter-binding assay was used to calculate the K_d_s of the mutated AC-I motifs (Table 4). Binding diminished when a mutation was present in the first three nucleotides of the AC-I motif (K_d_>5.00E^−06 ^M); however, when a mutation is present in the last three nucleotides of the AC-I motif, the binding is reduced but not completely abolished (Table 4). The relative binding affinities of recombinant *At*MYB61 protein to CASTing targets and mutated motifs were validated by EMSAs (Figure 2AB). EMSAs were conducted at a protein concentration of 5×10^−08 ^M because this was the protein concentration at which not all the targets reached their binding max as determined by nitrocellulose filter-binding assay (Figure 2AB, Table S1). This enabled detection of differential binding via EMSAs.

**Table 3:** Dissociation constants (K_d_) in mol/L and associated errors of CASTing targets.

	Kd	Error
**ACCTAC**	9.12E-09	3.11E-09
**ACCAAT**	1.21E-08	3.42E-09
**ACCAAA**	1.68E-08	4.07E-09
**ACCATA**	1.83E-08	5.06E-09
**ACCAAC**	7.37E-08	1.53E-08
**ACCACA**	8.08E-08	6.93E-09
**ACCACC**	6.90E-07	2.27E-08
**NBS**	>5.00E-06	

Relative binding affinities of the CASTing targets to *At*MYB61 were determined by a nitrocellulose filter-binding assay. The relative binding affinities were used to determine the dissociation constants of the CASTing targets by GRAFIT program which linearised the nonlinear regression via scatchard plots to calculate the point at which half of the ligand was bound to *At*MYB61. ACCTAC bound with the greatest affinity to *At*MYB61. NBS or non-binding site did not bind to recombinant *At*MYB61.

**Table 4:** Dissociation constants (K_d_) in mol/L and associated errors of mutated ACCTAC (AC1 element) sequences.

	Kd	Error
**ACCTAC**	9.12E-09	3.11E-09
**GCCTAC**	>5.00E-06	
**AGCTAC**	>5.00E-06	
**ACGTAC**	>5.00E-06	
**ACCGAC**	7.19E-07	2.12E-07
**ACCTGC**	7.97E-08	1.83E-08
**ACCTAG**	5.60E-08	5.09E-09

A guanine nucleotide was inserted one nucleotide at a time and shifted along the AC1 motif. Relative binding affinities of the mutated AC1 elements to *At*MYB61 were determined by a nitrocellulose filter-binding assay. The relative binding affinities were used to determine the dissociation constants of the CASTing targets by GRAFIT program which linearised the nonlinear regression via scatchard plots to calculate the point at which half of the ligand was bound to *At*MYB61. Underlined bases correspond to a substituted guanine.

*At*MYB61 bound its preferred target AC-I (ACCTAC) with a binding constant of 9.12E^−09 ^M (Table 3), which is similar to the tight binding of the vertebrate c-MYB R2R3 domain to the MYB binding site ((T/C)AAC(G/T)G(A/C/T)(A/C/T)) (binding constant = 1.5E^−09 ^M±28% ) [46], [47]. Tanikawa *et al.* found that AACG nucleotides in the MYBSI binding site were critical for binding [47]. The second adenine, fourth cytosine, and sixth guanine were particularly important in determining binding specificity. If any of these core nucleotides were mutated, binding affinity decreased by greater than 500 fold. The third adenine was not as crucial - if it was mutated, the binding affinity would be decreased up to 15 fold. Consistent with this, *At*MYB61 had a set of core recognition nucleotides – ACC – that could not be mutated without abolishing binding (Figure 2b, Table 4). Moreover, mutation of the latter half of the binding site, occurring at residues TAC, reduced binding but did not abolish it completely.

### The Affinity of *At*MYB61 to Specific Target DNA Sequences was Predicted by Molecular Interactions Determined *in Silico*

**Figure 3:**
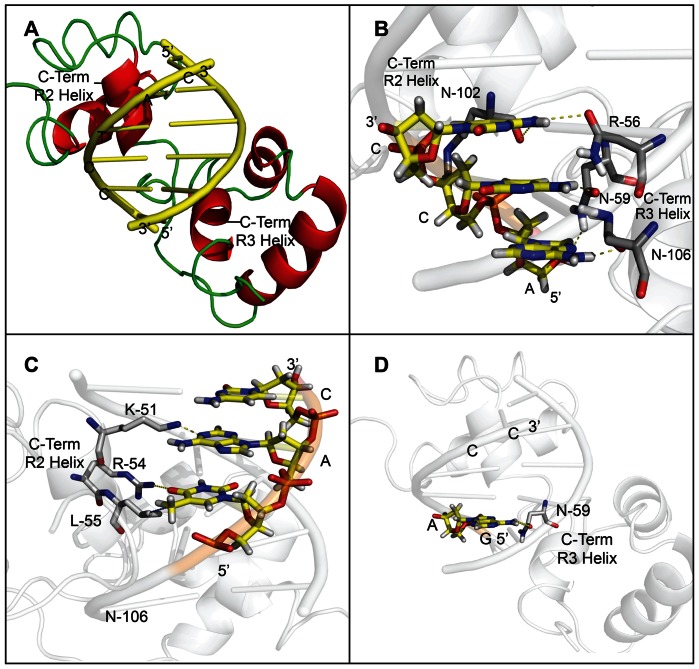
Molecular modelling of *At*MYB61 with target sequences confirm binding preferences determined by nitrocellulose filter-binding assays and EMSAs. (A) Pymol models of ACCTAC motif docked into the binding site of *At*MYB61. Molecular modelling was completed by using the online program PHYRE (Protein Homology/analogY Recognition Engine) to predict a crystal structure of *At*MYB61 using homology to the c-MYB DNA binding domain. The PBD (protein data bank) file recovered from the PHYRE analysis was used to superimpose the predicted *At*MYB61 crystal structure with the c-MYB crystal structure using DaliLite. Using Pymol the 3D sequence model – ACCTAC – was docked into the predicted binding sites of *At*MYB61. The AC1 element model is displayed in yellow, the loop secondary structure of *At*MYB61 inferred model is displayed in green, and the helix secondary structure of *At*MYB61 inferred model is displayed in red. (B) Model of *At*MYB61 binding site with the first three ACC nucleotides in the ACCTAC sequence determines that these nucleotides are essential for binding. The AC1 (ACCTAC) nucleotide model was docked into the predicted binding site of *At*MYB61. The specific hydrogen bonding between the amino acids of *At*MYB61 binding site to the ACC nucleotides of AC1 were predicted by Pymol and listed as follows: asparagine-59 (R3 helix) hydrogen to adenine-1 nitrogen; asparagine-106 (R3 helix) oxygen to adenine-1 hydrogen; asparagine-59 (R3 helix) oxygen to cytosine-2 hydrogen; asparagine-102 (R3 helix) oxygen to cytosine-3 hydrogen; and arginine-56 (R2 helix) oxygen to cytosine-3 hydrogen. This confirms binding data determined by the nitrocellulose filter-binding assay and EMSAs, iterating that the ACC motif is the core recognition motif of *At*MYB61. (C) Model of *At*MYB61 binding site with the TAC nucleotides in the ACCTAC sequence determine that these nucleotides are less essential for binding. The AC1 (ACCTAC) nucleotide models were docked into the predicted binding sites of *At*MYB61. The molecular interactions between the amino acids of *At*MYB61 binding site and the TAC nucleotides of AC1 were analyzed by Pymol and are listed as follows: leucine-55 (R2 helix) methyl group was predicted to form a non-polar bond with thymidine-4 methyl group; Arginine-54 (R2 helix) hydrogen was predicted to form a hydrogen bound with thymidine-4 oxygen; lysine-51 (R2 helix) hydrogen was predicted to form a hydrogen bound with adenine-5 nitrogen; and cytosine-6 remained unbound in the model. (D) Model of *At*MYB61 binding site with non-binding site (GAGACC) predicts that this motif is not recognised by *At*MYB61. The non binding site model was docked into *At*MYB61 binding site via Pymol and hydrogen bonding was analyzed. Only one hydrogen bond was predicted between *At*MYB61 asparagine-59 (R3 helix) oxygen and the non-binding site adenine-2 hydrogen. Yellow dashed lines indicate hydrogen bonding established by Pymol program, and blue dashed lines indicate non-polar interactions.

Computational analysis of the 3-dimensional structure of the N-terminal DNA-binding region of *At*MYB61 was conducted in order to validate the role of this domain in sequence-specific binding. Previously, the structure of the N-terminal DNA-binding domain of animal c-MYB bound to its DNA consensus motif (AACNG) was solved by heteronuclear multidimensional NMR [31]. Animal c-MYB DNA-binding region contains a conserved R2R3-MYB domain that exhibits high similarity to plant R2R3-MYB DNA binding domains. This NMR structure was used as a template to model the structure of *At*MYB61. The AC-I (ACCTAC) and NBS (GAGACC) nucleotide models were then docked into the predicted binding sites of the *At*MYB61 model (Figure 3).

Based on the model of *At*MYB61, the molecular interactions shared between the binding sites of *At*MYB61 to its targets supported *in vitro* binding data (Figure 3BCD). For example, there were more hydrogen bonds shared between *At*MYB61 DNA-binding domain and AC-I compared to NBS (Figure 3BCD). Based on the model of *At*MYB61 bound to AC-I, several specific intermolecular interactions are predicted to create binding specificity. These include hydrogen bonds between the following residues: asparagine-59 (R3 helix) of *At*MYB61 with adenine-1 nitrogen of AC-I; asparagine-106 (R3 helix) oxygen of *At*MYB61 with adenine-1 hydrogen of AC-I; asparagine-59 (R3 helix) oxygen of *At*MYB61 with cytosine-2 hydrogen of AC-I; asparagine-102 (R3 helix) oxygen of *At*MYB61 with cytosine-3 hydrogen of AC-I; arginine-56 (R2 helix) oxygen of *At*MYB61 with cytosine-3 hydrogen of AC-I; arginine-54 (R2 helix) hydrogen of *At*MYB61 with thymidine-4 oxygen of AC-I; and, lysine-51 (R2 helix) of *At*MYB61 with adenine-5 nitrogen of AC-I. The leucine-55 (R2 helix) methyl group of *At*MYB61 is predicted to form a non-polar bond with thymidine-4 methyl group of AC-I. Cytosine-6 remained unbound in the model. In comparison, the NBS model had only one hydrogen bond present, involving asparagine-59 (R3 helix) oxygen of *At*MYB61 with adenine-2 hydrogen of AC-I.

### The Affinity of *At*MYB61 to Specific Target DNA Sequences did not Correlate with *At*MYB61-driven Transcriptional Activation with each of the Target Sequences

**Figure 4:**
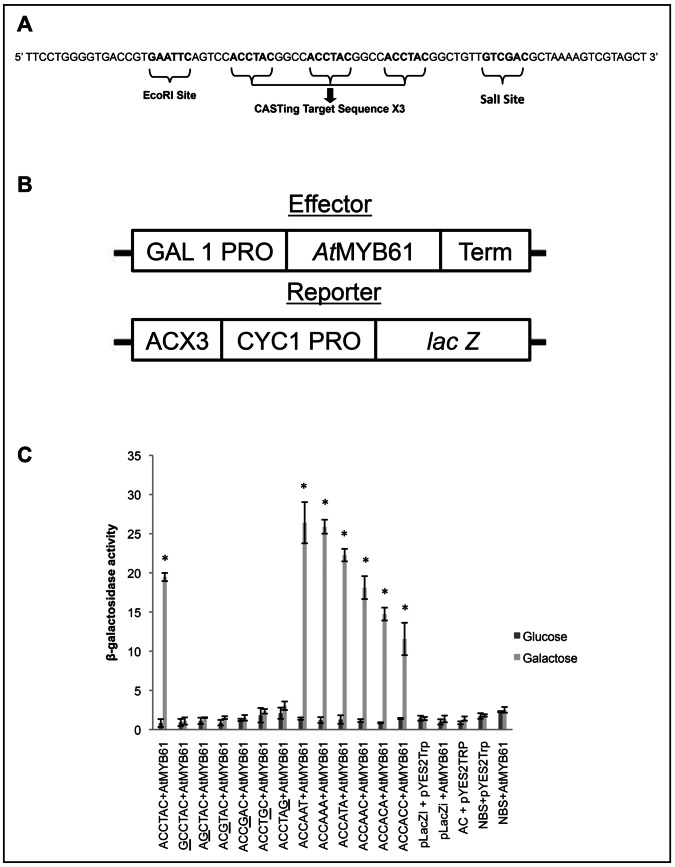
*At*MYB61-mediated activation of promoter activity in Saccharomyces cerevisiae in an AC dependent fashion. (A) The sequence of the oligonucleotides cloned into the reporter vector using EcoRI and SalI sites. Each AC element or mutated ACI element is triplicated within the segment. (B) Schematic representations of the Effector (pYES2TRP::AtMYB61) and Reporter (pLacZi::AC) constructs used in this assay (CYC1: minimal yeast promoter). (C) Quantitative analysis of β-galactosidase activity in yeast after induction. The measurements in liquid assay were made from three biological independent replicates. Activation of artificial genes comprising a minimal CYC1 promoter fused to a tandem AC element or mutated ACI element upstream of the lacZ gene by AtMYB61 protein, upon growth of the yeast in galactose (light grey bars), gave rise to β-galactosidase activity that was significantly greater than the controls, as determined by analysis of variance (P<0.005); including each vector alone, or both together after growth on non-inducing glucose (dark grey bars). Error bars represent standard deviations. *indicates statistically significant, P<0.005, determined by t-test. Underlined bases corresponds to a substituted guanine.

Previous studies have shown that *At*MYB61 protein is sufficient to drive transcription in yeast from promoter sequences that contain AC elements [34]. Consequently, yeast transcriptional activation assays were used to determine the relationship between *At*MYB61 affinity to specific DNA sequences and its capacity to drive transcription (Figure 4). Reporter constructs comprised the coding sequence for β-galactosidase under the control of the yeast minimal CYC1 promoter fused to triple repeats of a given CASTing target or a mutated AC-I motif (Figure 4). The minimal CYC1 promoter is unable to support transcription, so reporter expression would be contingent on the capacity of *At*MYB61 to bind to the fused motifs, which would function as gene regulatory sequences. The expression of *At*MYB61 was under the control of the galactose-inducible GAL1 promoter. As determined by the quantification of β-galactosidase activity, when *At*MYB61 protein was induced by galactose, the protein was able to activate transcription from the CASTing target sequences but not from the mutated AC-I elements (Figure 4). The extent of transcriptional activation varied for each CASTing target (Figure 4C). Notably, CASTing target sequences ACCATA, ACCAAT, and ACCAAA supported greater amounts of β-galactosidase induction relative to the AC-I element, which bound with the greatest affinity to *At*MYB61 (Figure 4C).

Previously, R2R3-MYB proteins have been shown to bind to AC elements and activate transcription in yeast and *in planta*; however, these studies did not correlate binding affinity with ability to activate transcription [8], [15], [17], [28]. Yeast activation assays determined that the affinity of *At*MYB61 to specific target DNA sequences did not correlate with *At*MYB61-driven transcriptional activation with each of the target sequences. This is not surprising given the multitude of studies that have observed that *in vitro* binding and endogenous transcriptional regulation frequently disagree [48], [49], [50], [51], [52]. Consistent with this are results obtained using the glucocorticoid receptor (GR), where no correlation between *in vitro* binding affinities and *in vivo* transcriptional activities was observed [53]. GR target sequences, differing by as little as a single nucleotide, differentially affected GR DNA binding and transcriptional activity, with no correlation between these parameters. Similarly, binding affinity of *At*MYB61 to specific target DNA sequences did not correlate with *At*MYB61-driven transcriptional activation with each of the target sequences. It may be that conformation of *At*MYB61 changes when binding to a specific DNA sequence, altering its ability to activate transcription.

### CASTing Target Sequences were Found in the Promoter Regions of Three Putative Direct Downstream Targets of *At*MYB61

Previous experiments predicted three putative direct downstream target genes of *At*MYB61 [34]. The predicted targets of *At*MYB61 were determined by a two-stage comparative transcriptome analysis. This transcriptome analysis entailed identification and comparison of genes whose transcript abundance was modulated by differences in *At*MYB61 activity, relative to those genes whose transcript abundance profiles paralleled *AtMYB61* across development and in different organs. In the first stage of the analysis, publicly available, complete *Arabidopsis* transcriptome microarray data were used to identify those genes sharing the same transcript abundance profile as *AtMYB61* across multiple stages of development. The second stage of transcriptome analysis identified genes whose transcript abundance was influenced by the presence or absence of *At*MYB61. A complete transcriptome microarray dataset was generated using WT, *myb61* loss-of-function mutants and *35S::MYB61* gain-of-function transgenic plants, enabling comparison of the impact of *At*MYB61 on transcriptome activity. Genes were identified in this dataset whose transcript abundance was contingent on the relative abundance of *AtMYB61*. Both gene lists were then compared to identify genes that were most likely direct targets of *At*MYB61. Three genes were identified in the intersection set. They encode the following gene products: a KNOTTED1-like transcription factor (KNAT7, At1g62990); a Caffeoyl-CoA 3-O-methyltransferase (CCoAOMT7, At4g26220); and a pectin-methylesterase (PME, At2g45220). In keeping with their role as putative *At*MYB61 targets, loss-of-function mutants corresponding to each of the genes phenocopy aspects of the *myb61* loss-of-function mutant phenotype.

**Figure 5:**
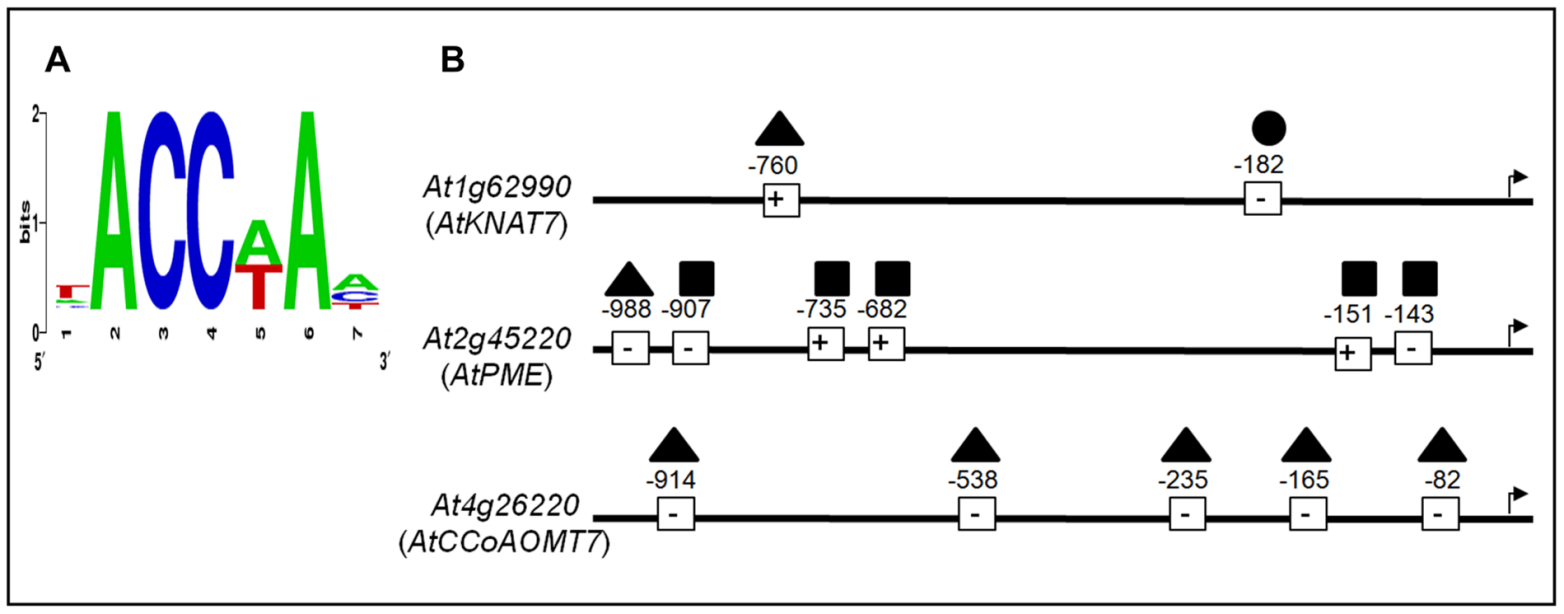
Sequences recovered from the CASTing assay were over-represented in all three promoter regions of predicted direct downstream targets of AtMYB61. (A) Sequence logo of the over-represented motifs in the promoter sequences of the three target genes of *At*MYB61, as determined by the Promomer algorithm [54] (average = 2.9; *Z*-score = 13; significance = 0.001). Right: Schematic representation of the promoter regions of the three putative *At*MYB61 downstream target genes. (B) The three putative AtMYB61 direct downstream target genes,, KNOTTED1-like transcription factor (KNAT7, At1g62990); Caffeoyl-CoA 3-O-methyltransferase (CCoAOMT7, At4g26220), and pectin-methylesterase (PME, At2g45220), were identified by Romano *et al* (33). 1000 bp upstream regulatory regions were examined of the three genes. +/− indicate the orientation of CASTing target sequences relative to the sense coding strand; whereas, numbers indicate the position of these motifs relative to the putative transcriptional start (indicated by an arrow). Triangle represents ACCAAA, square represents ACCAAT, and circle represents ACCATA.

The CASTing targets were identified in the 1000 bp 5′ non-coding regions of the three putative direct target genes and were determined by algorithm-based screening to be statistically over-represented (Figure 5). *At*MYB61 bound to the 5′ gene regulatory sequences of all three putative direct target genes in an AC dependent manner [34]. These data support the hypothesis that *At*MYB61 binds to AC elements in a distinct set of target genes to modify gene expression.

### Conclusion

Despite the size and importance of the plant R2R3-MYB family of transcriptional regulators, little is known about the molecular functioning of given family members. The work described herein casts greater light on the interaction between an R2R3-MYB family member and its cognate DNA targets. The findings support the hypothesis that *At*MYB61 is recruited to target genes via its interactions with a set of unique sequences, and thereby modifies gene expression. Surprisingly, the affinity of AtMYB61 to specific target DNA sequences did not correlate with *At*MYB61-driven transcriptional activation with each of the target sequences, suggesting that the conformation of *At*MYB61 may be altered allosterically when bound to specific target sequences. These findings point to additional complexities in the regulation of plant gene expression, and argue for the need for greater exploration of the molecular intricacies involved in the interactions between plant transcription factors and their DNA targets.

## Supporting Information

Figure S1 ***At*** **MYB61 antibody generation and validation.** (A) Amino acid sequence similarity of *At*MYB61 along with its closest family member *At*MYB50. The two proteins have conserved N-terminal amino acid sequences but unique C-terminal domains, which was the domain selected to generate *At*MYB61 antibodies against (highlighted region). (B) A chemiluminescent western blot validate anti-*At*MYB61 antibody specificity. Lanes correspond to full-length recombinant *At*MYB61 protein (Lane 1), of antibody alone (Lane 2), and *At*MYB61 recombinant protein immunoprecipitated with pre-immune serum (Lane 3) and with *At*MYB61-specific antiserum (Lane 4). Western blot was done with 1∶20000 dilution of immune serum. Western blot shows greater quantities of *At*MYB61 protein eluted from the Magnetic Dynabeads Protein G antibody complex compared to the Magnetic Dynabeads Protein G pre-immune serum complex, showing that the immunoprecipitation was successful.
(TIF)

Table S1 **Relative binding of CASTing targets and mutated AC-I sequences to** ***At*** **MYB61.**
(DOC)

## References

[1] Arabidopsis Genome I (2000) Analysis of the genome sequence of the flowering plant Arabidopsis thaliana. Nature 408: 796–815. 11130711 10.1038/35048692

[2] Dubos C, Stracke R, Grotewold E, Weisshaar B, Martin C, et al (2010) MYB transcription factors in Arabidopsis. Trends in Plant Science 15: 573–581. 20674465 10.1016/j.tplants.2010.06.005

[3] Martin C, PazAres J (1997) MYB transcription factors in plants. Trends in Genetics 13: 67–73. 9055608 10.1016/s0168-9525(96)10049-4

[4] Riechmann JL, Heard J, Martin G, Reuber L, Jiang CZ, et al (2000) Arabidopsis transcription factors: Genome-wide comparative analysis among eukaryotes. Science 290: 2105–2110. 11118137 10.1126/science.290.5499.2105

[5] Stracke R, Werber M, Weisshaar B (2001) The R2R3-MYB gene family in Arabidopsis thaliana. Current Opinion in Plant Biology 4: 447–456. 11597504 10.1016/s1369-5266(00)00199-0

[6] Tombuloglu H, Kekec G, Sakcali MS, Unver T (2013) Transcriptome-wide identification of R2R3-MYB transcription factors in barley with their boron responsive expression analysis. Mol Genet Genomics 288: 141–155. 23539153 10.1007/s00438-013-0740-1

[7] Rosinski JA, Atchley WR (1998) Molecular evolution of the Myb family of transcription factors: Evidence for polyphyletic origin. Journal of Molecular Evolution 46: 74–83. 9419227 10.1007/pl00006285

[8] Gomez-Maldonado J, Avila C, de la Torre F, Canas R, Canovas FM, et al (2004) Functional interactions between a glutamine synthetase promoter and MYB proteins. Plant Journal 39: 513–526. 15272871 10.1111/j.1365-313X.2004.02153.x

[9] Glover BJ, Perez-Rodriguez M, Martin C (1998) Development of several epidermal cell types can be specified by the same MYB-related plant transcription factor. Development 125: 3497–3508. 9693152 10.1242/dev.125.17.3497

[10] Jin HL, Martin C (1999) Multifunctionality and diversity within the plant MYB-gene family. Plant Molecular Biology 41: 577–585. 10645718 10.1023/a:1006319732410

[11] Lipsick JS (1996) One billion years of Myb. Oncogene 13: 223–235. 8710361

[12] Martin C, Bhatt K, Baumann K, Jin H, Zachgo S, et al (2002) The mechanics of cell fate determination in petals. Philosophical Transactions of the Royal Society of London Series B-Biological Sciences 357: 809–813. 10.1098/rstb.2002.1089 PMC1692987 12079676

[13] Liang YK, Dubos C, Dodd IC, Holroyd GH, Hetherington AM, et al (2005) AtMYB61, an R2R3-MYB transcription factor controlling stomatal aperture in Arabidopsis thaliana. Current Biology 15: 1201–1206. 16005292 10.1016/j.cub.2005.06.041

[14] Newman LJ, Perazza DE, Juda L, Campbell MM (2004) Involvement of the R2R3-MYB, AtMYB61, in the ectopic lignification and dark-photomorphogenic components of the det3 mutant phenotype. Plant Journal 37: 239–250. 14690508 10.1046/j.1365-313x.2003.01953.x

[15] Patzlaff A, Newman LJ, Dubos C, Whetten R, Smith C, et al (2003) Characterisation of PtMYB1, an R2R3-MYB from pine xylem. Plant Molecular Biology 53: 597–608. 15010621 10.1023/B:PLAN.0000019066.07933.d6

[16] Prouse MB, Campbell MM (2012) The interaction between MYB proteins and their target DNA binding sites. Biochimica Et Biophysica Acta-Gene Regulatory Mechanisms 1819: 67–77. 10.1016/j.bbagrm.2011.10.010 22067744

[17] Patzlaff A, McInnis S, Courtenay A, Surman C, Newman LJ, et al (2003) Characterisation of a pine MYB that regulates lignification. Plant Journal 36: 743–754. 14675440 10.1046/j.1365-313x.2003.01916.x

[18] Hatton D, Sablowski R, Yung MH, Smith C, Schuch W, et al (1995) 2 Classes of cis sequences contribute to tissue-specific expression of a PAL2 promtoer in transgenic tobacco. Plant Journal 7: 859–876. 7599647 10.1046/j.1365-313x.1995.07060859.x

[19] BellLelong DA, Cusumano JC, Meyer K, Chapple C (1997) Cinnamate-4-hydroxylase expression in Arabidopsis - Regulation in response to development and the environment. Plant Physiology 113: 729–738. 9085570 10.1104/pp.113.3.729 PMC158190

[20] Hauffe KD, Lee SP, Subramaniam R, Douglas CJ (1993) Combinatorial interactions between positive and negative cis-acting elements control spatial patterns of 4CL-1 expression in transgenic tobacco Plant Journal. 4: 235–253. 10.1046/j.1365-313x.1993.04020235.x 8220481

[21] Joos HJ, Hahlbrock K (1992) Phenylalanine ammonia-lyase in potato (Solanum-tuberosum L) - genomic complexity, structural comparison of 2 selected genes and modes of expression European Journal of Biochemistry. 204: 621–629. 10.1111/j.1432-1033.1992.tb16675.x 1541277

[22] Lacombe E, Van Doorsselaere J, Boerjan W, Boudet AM, Grima-Pettenati J (2000) Characterization of cis-elements required for vascular expression of the Cinnamoyl CoA Reductase gene and for protein-DNA complex formation. Plant Journal 23: 663–676. 10972892 10.1046/j.1365-313x.2000.00838.x

[23] Lauvergeat V, Rech P, Jauneau A, Guez C, Coutos-Thevenot P, et al (2002) The vascular expression pattern directed by the Eucalyptus gunnii cinnamyl alcohol dehydrogenase EgCAD2 promoter is conserved among woody and herbaceous plant species. Plant Molecular Biology 50: 497–509. 12369625 10.1023/a:1019817913604

[24] Leyva A, Liang XW, Pintortoro JA, Dixon RA, Lamb CJ (1992) Cis-element combinations determine phenylalanine ammonia-lyase gene tissue-specific expression patterns. Plant Cell 4: 263–271. 1498596 10.1105/tpc.4.3.263 PMC160127

[25] Logemann E, Parniske M, Hahlbrock K (1995) Modes of expression and common structural features of the complete phenylalanine ammonia-lyase gene family in parsley. Proceedings of the National Academy of Sciences of the United States of America 92: 5905–5909. 7597051 10.1073/pnas.92.13.5905 PMC41610

[26] Lois R, Dietrich A, Hahlbrock K, Schulz W (1989) A phenylalanine ammonia-lyase gene from parsley - structure, regulation and identification of elicitor and light responsive cis-acting elements. Embo Journal 8: 1641–1648. 2767049 10.1002/j.1460-2075.1989.tb03554.x PMC401003

[27] Seguin A, Laible G, Leyva A, Dixon RA, Lamb CJ (1997) Characterization of a gene encoding a DNA-binding protein that interacts in vitro with vascular specific cis elements of the phenylalanine ammonia-lyase promoter. Plant Molecular Biology 35: 281–291. 9349252 10.1023/a:1005853404242

[28] Jin HL, Cominelli E, Bailey P, Parr A, Mehrtens F, et al (2000) Transcriptional repression by AtMYB4 controls production of UV-protecting sunscreens in Arabidopsis. Embo Journal 19: 6150–6161. 11080161 10.1093/emboj/19.22.6150 PMC305818

[29] Ogata K, Kanai H, Inoue T, Sekikawa A, Sasaki M, et al. (1993) Solution structures of Myb DNA-binding domain and its complex with DNA. Nucleic acids symposium series: 201–202. 8247769

[30] Ogata K, Morikawa S, Nakamura H, Hojo H, Yoshimura S, et al (1995) Comparison of the free and DNA-complexed forms of the DNA-binding domain from c-Myb. Nature Structural Biology 2: 309–320. 7796266 10.1038/nsb0495-309

[31] Ogata K, Morikawa S, Nakamura H, Sekikawa A, Inoue T, et al (1994) Solution structure of a specific DNA complex of the Myb DNA-binding domain with cooperative recognition helices. Cell 79: 639–648. 7954830 10.1016/0092-8674(94)90549-5

[32] Tahirov TH, Sasaki M, Inoue-Bungo T, Fujikawa A, Sato K, et al (2001) Crystals of ternary protein-DNA complexes composed of DNA-binding domains of c-Myb or v-Myb, C/EBP alpha or C/EBP beta and tom-1A promoter fragment. Acta Crystallographica Section D-Biological Crystallography 57: 1655–1658. 10.1107/s0907444901011982 11679735

[33] Tahirov TH, Sato K, Ichikawa-Iwata E, Sasaki M, Inoue-Bungo T, et al (2002) Mechanism of c-Myb-C/EBP beta cooperation from separated sites on a promoter. Cell 108: 57–70. 11792321 10.1016/s0092-8674(01)00636-5

[34] Romano JM, Dubos C, Prouse MB, Wilkins O, Hong H, et al (2012) AtMYB61, an R2R3-MYB transcription factor, functions as a pleiotropic regulator via a small gene network. New Phytologist 195: 774–786. 22708996 10.1111/j.1469-8137.2012.04201.x

[35] Dubos C, Willment J, Huggins D, Grant GH, Campbell MM (2005) Kanamycin reveals the role played by glutamate receptors in shaping plant resource allocation. Plant Journal 43: 348–355. 16045471 10.1111/j.1365-313X.2005.02458.x

[36] Penfield S, Meissner RC, Shoue DA, Carpita NC, Bevan MW (2001) MYB61 is required for mucilage deposition and extrusion in the Arabidopsis seed coat. Plant Cell 13: 2777–2791. 11752387 10.1105/tpc.010265 PMC139488

[37] Wright WE, Binder M, Funk W (1991) Cyclic amplification and selection of targets (CASTing) for the myogenin consensus binding site. Molecular and Cellular Biology 11: 4104–4110. 1649388 10.1128/mcb.11.8.4104-4110.1991 PMC361222

[38] Harlow E, and Lane D. (1988) Antibodies: A Laboratory Manual. Cold Spring Harbor NY. Cold Spring Harbor Laboratory Press.

[39] Bailey TL, Williams N, Misleh C, Li WW (2006) MEME: discovering and analyzing DNA and protein sequence motifs. Nucleic Acids Research 34: W369–W373. 16845028 10.1093/nar/gkl198 PMC1538909

[40] Hall KB, and Kranz J.K. (2008) Nitrocellulose Filter Binding for Determination of Dissociation Constants. In RNA Protein Interaction Protocols Humana Press: 105–114. 10.1385/1-59259-676-2:105 10549518

[41] McDonnell AV, Jiang T, Keating AE, Berger B (2006) Paircoil2: improved prediction of coiled coils from sequence. Bioinformatics 22: 356–358. 16317077 10.1093/bioinformatics/bti797

[42] Holm L, Park J (2000) DaliLite workbench for protein structure comparison. Bioinformatics 16: 566–567. 10980157 10.1093/bioinformatics/16.6.566

[43] Seeliger D, de Groot BL (2010) Ligand docking and binding site analysis with PyMOL and Autodock/Vina. Journal of Computer-Aided Molecular Design 24: 417–422. 20401516 10.1007/s10822-010-9352-6 PMC2881210

[44] DeLano WL (2002) The PyMOL Molecular Graphics System DeLano Scientific. http://wwwpymolorg.

[45] Goicoechea M, Lacombe E, Legay S, Mihaljevic S, Rech P, et al (2005) EgMYB2, a new transcriptional activator from Eucalyptus xylem, regulates secondary cell wall formation and lignin biosynthesis. Plant Journal 43: 553–567. 16098109 10.1111/j.1365-313X.2005.02480.x

[46] Ebneth A, Schweers O, Thole H, Fagin U, Urbanke C, et al (1994) Biophysical characterization of the c-Myb DNA-binding domain. Biochemistry 33: 14586–14593. 7981220 10.1021/bi00252a026

[47] Tanikawa J, Yasukawa T, Enari M, Ogata K, Nishimura Y, et al (1993) Recognition of specific DNA sequences by the c-myb protooncogene product: role of three repeat units in the DNA-binding domain. Proceedings of the National Academy of Sciences of the United States of America 90: 9320–9324. 8415700 10.1073/pnas.90.20.9320 PMC47559

[48] Fuxreiter M, Simon I, Bondos S (2011) Dynamic protein-DNA recognition: beyond what can be seen. Trends in Biochemical Sciences 36: 415–423. 21620710 10.1016/j.tibs.2011.04.006

[49] Lefstin JA, Yamamoto KR (1998) Allosteric effects of DNA on transcriptional regulators. Nature 392: 885–888. 9582068 10.1038/31860

[50] Massie CE, Mills IG (2008) ChIPping away at gene regulation. Embo Reports 9: 337–343. 18379585 10.1038/embor.2008.44 PMC2288763

[51] Rohs R, Jin XS, West SM, Joshi R, Honig B, et al. (2010) Origins of Specificity in Protein-DNA Recognition. In: Kornberg RD, Raetz CRH, Rothman JE, Thorner JW, editors. Annual Review of Biochemistry, Vol 79. Palo Alto: Annual Reviews. 233–269. 10.1146/annurev-biochem-060408-091030 PMC3285485 20334529

[52] Wang J, Lu J, Gu G, Liu Y (2011) In vitro DNA-binding profile of transcription factors: methods and new insights. Journal of Endocrinology 210: 15–27. 21389103 10.1530/JOE-11-0010

[53] Meijsing SH, Pufall MA, So AY, Bates DL, Chen L, et al (2009) DNA Binding Site Sequence Directs Glucocorticoid Receptor Structure and Activity. Science 324: 407–410. 19372434 10.1126/science.1164265 PMC2777810

[54] Toufighi K, Brady SM, Austin R, Ly E, Provart NJ (2005) The Botany Array Resource: e-Northerns, Expression Angling, and Promoter analyses. Plant Journal 43: 153–163. 15960624 10.1111/j.1365-313X.2005.02437.x

