# Supplementary figures and images for: Interactions between the R2R3-MYB Transcription Factor, AtMYB61, and Target DNA Binding Sites
Source: PLoS One. 2013 May 31;8(5):e65132. doi: 10.1371/journal.pone.0065132 (PMC3669277; doi:10.1371/journal.pone.0065132)

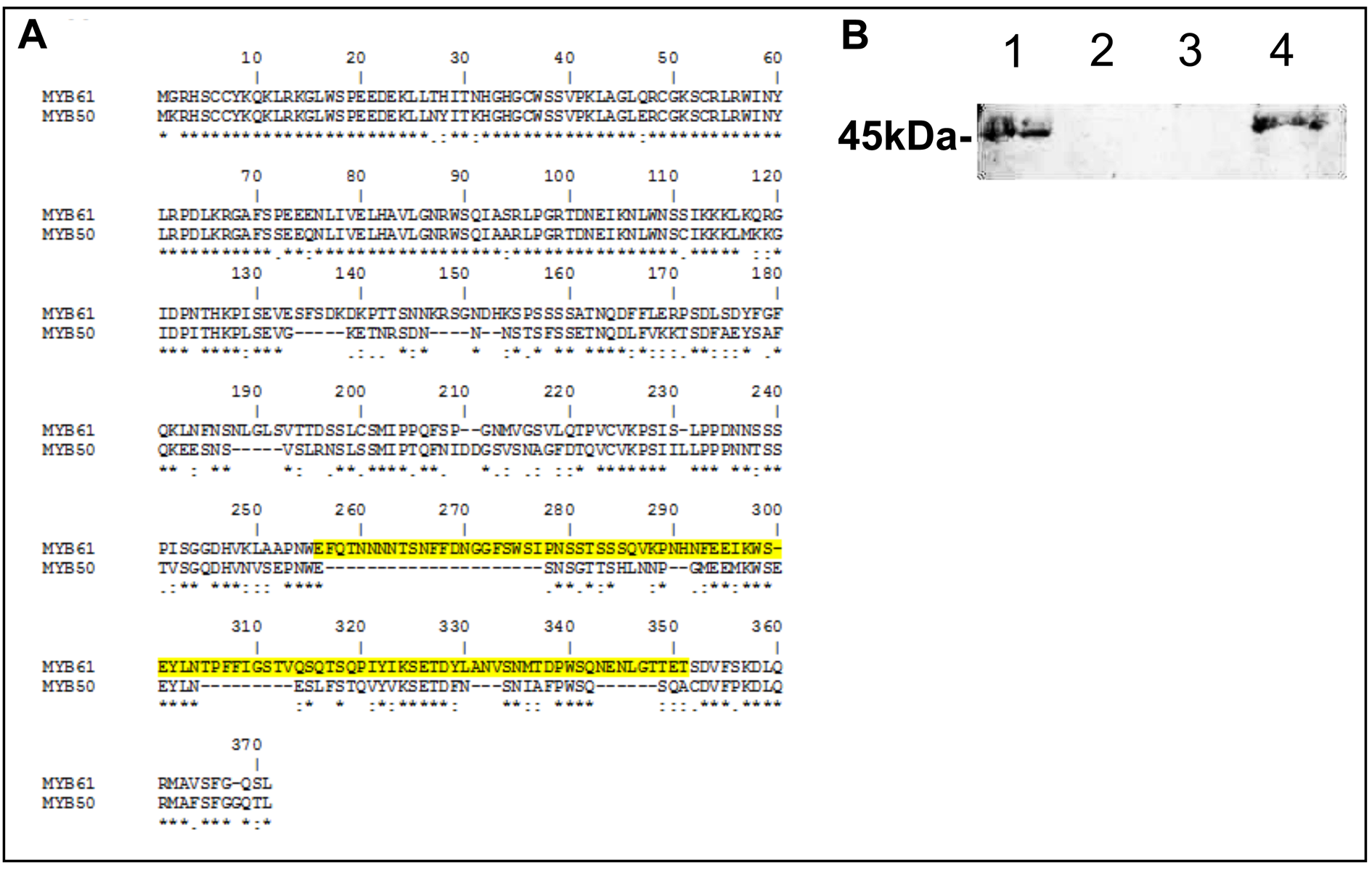

Supplement: Figure S1 — At MYB61 antibody generation and validation. (A) Amino acid sequence similarity of AtMYB61 along with its closest family member AtMYB50. The two proteins have conserved N-terminal amino acid sequences but unique C-terminal domains, which was the domain selected to generate AtMYB61 antibodies against (highlighted region). (B) A chemiluminescent western blot validate anti-AtMYB61 antibody specificity. Lanes correspond to full-length recombinant AtMYB61 protein (Lane 1), of antibody alone (Lane 2), and AtMYB61 recombinant protein immunoprecipitated with pre-immune serum (Lane 3) and with AtMYB61-specific antiserum (Lane 4). Western blot was done with 1∶20000 dilution of immune serum. Western blot shows greater quantities of AtMYB61 protein eluted from the Magnetic Dynabeads Protein G antibody complex compared to the Magnetic Dynabeads Protein G pre-immune serum complex, showing that the immunoprecipitation was successful. (TIF) [file pone.0065132.s001.tif]
