# Supplementary material for: Interactions between the R2R3-MYB Transcription Factor, AtMYB61, and Target DNA Binding Sites
Source: PLoS One. 2013 May 31;8(5):e65132. doi: 10.1371/journal.pone.0065132 (PMC3669277; doi:10.1371/journal.pone.0065132)
Supplement: Table S1 — Relative binding of CASTing targets and mutated AC-I sequences to At MYB61. (DOC) [file pone.0065132.s002.doc]

| **Supplemental Table S1. Relative binding of CASTing targets and mutated AC-I sequences to *At*MYB61** | | | | | | | | | |
| --- | --- | --- | --- | --- | --- | --- | --- | --- | --- |
| **ACCAAC** | **1.00E-09** | **5.00E-09** | **1.00E-08** | **5.00E-08** | **1.00E-07** | **5.00E-07** | **1.00E-06** | **5.00E-06** | **Probe** |
| Trial 1 | 16203 | 20451 | 26456 | 111235 | 223153 | 310225 | 325212 | 335456 | 460122 |
| Trial 2 | 15145 | 18513 | 23513 | 92214 | 242153 | 315212 | 321021 | 324658 | 458213 |
| Trial 3 | 13142 | 19088 | 20578 | 114285 | 231026 | 288232 | 304666 | 307279 | 446521 |
| Average | 14830 | 19350 | 23515 | 105911 | 232110 | 304556 | 316966 | 322464 | 454952 |
| Binding | 0.0352182 | 0.045954 | 0.055845 | 0.251517 | 0.551214 | 0.723257 | 0.752728 | 0.765785 |  |
| **ACCACC** | **1.00E-09** | **5.00E-09** | **1.00E-08** | **5.00E-08** | **1.00E-07** | **5.00E-07** | **1.00E-06** | **5.00E-06** | **Probe** |
| Trial 1 | 32891 | 42654 | 47895 | 54112 | 112356 | 220167 | 328989 | 363354 | 481234 |
| Trial 2 | 33564 | 41258 | 45654 | 55333 | 111589 | 226896 | 322644 | 312578 | 495242 |
| Trial 3 | 38289 | 41124 | 46524 | 49992 | 117458 | 236446 | 328227 | 341592 | 475863 |
| Average | 34914 | 41678 | 46691 | 53145 | 113801 | 227836 | 326619 | 339174 | 484113 |
| Binding | 0.0774535 | 0.092459 | 0.103578 | 0.117897 | 0.252453 | 0.505425 | 0.724564 | 0.752415 |  |
| **ACCAAA** | **1.00E-09** | **5.00E-09** | **1.00E-08** | **5.00E-08** | **1.00E-07** | **5.00E-07** | **1.00E-06** | **5.00E-06** | **Probe** |
| Trial 1 | 26442 | 31996 | 176351 | 255645 | 319665 | 321348 | 345631 | 347562 | 496372 |
| Trial 2 | 25854 | 31254 | 169856 | 251335 | 314556 | 318964 | 342654 | 342556 | 489653 |
| Trial 3 | 22542 | 26978 | 141629 | 241274 | 310182 | 310808 | 331499 | 342380 | 481234 |
| Average | 24946 | 30076 | 162612 | 249418 | 314801 | 317040 | 339928 | 344166 | 489086.33 |
| Binding | 0.0551982 | 0.066549 | 0.359815 | 0.55189 | 0.696565 | 0.701519 | 0.752165 | 0.761542 |  |
| **ACCAAT** | **1.00E-09** | **5.00E-09** | **1.00E-08** | **5.00E-08** | **1.00E-07** | **5.00E-07** | **1.00E-06** | **5.00E-06** | **Probe** |
| Trial 1 | 20155 | 46254 | 211558 | 266242 | 312585 | 357456 | 354231 | 362645 | 487651 |
| Trial 2 | 18982 | 45335 | 208334 | 263423 | 311225 | 349978 | 344580 | 350024 | 480225 |
| Trial 3 | 19003 | 41241 | 196044 | 266540 | 305857 | 297827 | 292797 | 300952 | 479852 |
| Average | 19380 | 44276 | 205312 | 265401 | 309888 | 335086 | 330536 | 337873 | 482576 |
| Binding | 0.0431234 | 0.098522 | 0.456848 | 0.590556 | 0.689546 | 0.745615 | 0.735489 | 0.751816 |  |
| **ACCACA** | **1.00E-09** | **5.00E-09** | **1.00E-08** | **5.00E-08** | **1.00E-07** | **5.00E-07** | **1.00E-06** | **5.00E-06** | **Probe** |
| Trial 1 | 17245 | 24288 | 37524 | 126997 | 224568 | 308521 | 334568 | 337851 | 462254 |
| Trial 2 | 16670 | 22853 | 36293 | 124789 | 219895 | 305452 | 325586 | 329987 | 461235 |
| Trial 3 | 11815 | 24571 | 35957 | 117110 | 140819 | 296508 | 325909 | 318383 | 459978 |
| Average | 15243 | 23904 | 36591 | 122965 | 195093 | 303493 | 328687 | 328740 | 461155 |
| Binding | 0.0354852 | 0.055647 | 0.085182 | 0.286255 | 0.454165 | 0.706512 | 0.765162 | 0.765285 |  |

| **Table S1 continued** | | |  |  |  |  |  |  |  |
| --- | --- | --- | --- | --- | --- | --- | --- | --- | --- |
| **ACCATA** | **1.00E-09** | **5.00E-09** | **1.00E-08** | **5.00E-08** | **1.00E-07** | **5.00E-07** | **1.00E-06** | **5.00E-06** | **Probe** |
| Trial 1 | 21258 | 28269 | 171654 | 242588 | 324689 | 320115 | 345456 | 345571 | 475821 |
| Trial 2 | 20199 | 27855 | 169558 | 234560 | 311471 | 319524 | 339887 | 340129 | 474458 |
| Trial 3 | 18837 | 22493 | 154411 | 185371 | 266696 | 315799 | 309027 | 325918 | 468521 |
| Average | 20097 | 26205 | 165207 | 220839 | 300951 | 318479 | 331456 | 337206 | 472933 |
| Binding | 0.0456419 | 0.059512 | 0.375182 | 0.50152 | 0.683453 | 0.723257 | 0.752728 | 0.765785 |  |
| **ACCTAC** | **1.00E-09** | **5.00E-09** | **1.00E-08** | **5.00E-08** | **1.00E-07** | **5.00E-07** | **1.00E-06** | **5.00E-06** | **Probe** |
| Trial 1 | 42125 | 51226 | 251665 | 288968 | 332541 | 331224 | 361547 | 358702 | 489213 |
| Trial 2 | 40242 | 52874 | 248552 | 287110 | 325574 | 312123 | 358990 | 348873 | 486237 |
| Trial 3 | 37496 | 45365 | 232628 | 250710 | 288998 | 323556 | 288460 | 312273 | 480411 |
| Average | 39954 | 49821 | 244281 | 275596 | 315704 | 322300 | 336332 | 339949 | 485287 |
| Binding | 0.0882924 | 0.110098 | 0.539824 | 0.609024 | 0.697657 | 0.712234 | 0.743241 | 0.751234 |  |
| **GCCTAC** | **1.00E-09** | **5.00E-09** | **1.00E-08** | **5.00E-08** | **1.00E-07** | **5.00E-07** | **1.00E-06** | **5.00E-06** | **Probe** |
| Trial 1 | 13586 | 23558 | 25334 | 34135 | 55101 | 68951 | 62548 | 66352 | 478921 |
| Trial 2 | 11440 | 21040 | 22114 | 37526 | 57241 | 69524 | 60177 | 63874 | 476621 |
| Trial 3 | 5666 | 15369 | 22859 | 37855 | 50639 | 65307 | 54646 | 56274 | 465312 |
| Average | 10230 | 19989 | 23435 | 36505 | 54326 | 67927 | 59123 | 62166 | 473618 |
| Binding | 0.0232423 | 0.0454123 | 0.0532423 | 0.0829349 | 0.123423 | 0.154321 | 0.134321 | 0.141234 |  |
| **AGCTAC** | **1.00E-09** | **5.00E-09** | **2.00E+00** | **5.00E-08** | **1.00E-07** | **5.00E-07** | **1.00E-06** | **5.00E-06** | **Probe** |
| Trial 1 | 14448 | 18542 | 23868 | 38512 | 51224 | 85304 | 71452 | 74289 | 468621 |
| Trial 2 | 16273 | 17520 | 21264 | 37246 | 49254 | 82330 | 68871 | 72555 | 461255 |
| Trial 3 | 13322 | 10614 | 21663 | 29321 | 42945 | 81802 | 53817 | 68382 | 458913 |
| Average | 14680 | 15558 | 22264 | 35026 | 47807 | 83145 | 64713 | 71741 | 462929 |
| Binding | 0.0345132 | 0.0365768 | 0.0523423 | 0.0823432 | 0.11239 | 0.195465 | 0.152134 | 0.168657 |  |
| **ACGTAC** | **1.00E-09** | **5.00E-09** | **1.00E-08** | **5.00E-08** | **1.00E-07** | **5.00E-07** | **1.00E-06** | **5.00E-06** | **Probe** |
| Trial 1 | 27881 | 31456 | 44586 | 49853 | 78921 | 69889 | 81227 | 83556 | 498533 |
| Trial 2 | 26648 | 35213 | 41001 | 44571 | 69246 | 71526 | 82254 | 84470 | 491524 |
| Trial 3 | 22840 | 22499 | 32838 | 35768 | 78246 | 62399 | 71100 | 90315 | 489255 |
| Average | 25789 | 29722 | 39475 | 43397 | 75471 | 67937 | 78526 | 86113 | 493104 |
| Binding | 0.0565421 | 0.0651652 | 0.0865465 | 0.0951456 | 0.165465 | 0.148949 | 0.172165 | 0.188798 |  |

| **Table S1 Continued** | | |  |  |  |  |  |  |  |
| --- | --- | --- | --- | --- | --- | --- | --- | --- | --- |
| **ACCGAC** | **1.00E-09** | **5.00E-09** | **1.00E-08** | **5.00E-08** | **1.00E-07** | **5.00E-07** | **1.00E-06** | **5.00E-06** | **Probe** |
| Trial 1 | 8014 | 18520 | 43558 | 51201 | 63599 | 133563 | 262147 | 335864 | 475561 |
| Trial 2 | 4861 | 14332 | 41002 | 49664 | 54248 | 119211 | 246610 | 312247 | 472608 |
| Trial 3 | 3577 | 9157 | 43689 | 42203 | 49600 | 113920 | 210525 | 333435 | 467823 |
| Average | 5484 | 14003 | 42749 | 47689 | 55815 | 122231 | 239760 | 327182 | 471997 |
| Binding | 0.012591 | 0.03215 | 0.098156 | 0.109498 | 0.12816 | 0.280651 | 0.55051 | 0.75123 |  |
| **ACCTGC** | **1.00E-09** | **5.00E-09** | **1.00E-08** | **5.00E-08** | **1.00E-07** | **5.00E-07** | **1.00E-06** | **5.00E-06** | **Probe** |
| Trial 1 | 17265 | 34552 | 36521 | 92234 | 233458 | 290142 | 330121 | 388914 | 466258 |
| Trial 2 | 16998 | 32621 | 37229 | 88841 | 225895 | 289521 | 322449 | 311258 | 462135 |
| Trial 3 | 9756 | 34186 | 31294 | 94421 | 207276 | 256933 | 308889 | 269165 | 459532 |
| Average | 14673 | 33786 | 35014 | 91832 | 222209 | 278865 | 320486 | 323112 | 462641 |
| Binding | 0.034285 | 0.07895 | 0.081816 | 0.214575 | 0.51922 | 0.651598 | 0.74885 | 0.75499 |  |
| **ACCTAG** | **1.00E-09** | **5.00E-09** | **1.00E-08** | **5.00E-08** | **1.00E-07** | **5.00E-07** | **1.00E-06** | **5.00E-06** | **Probe** |
| Trial 1 | 18554 | 34858 | 44578 | 155441 | 225898 | 321580 | 335412 | 344521 | 465852 |
| Trial 2 | 15587 | 33654 | 41876 | 159872 | 241014 | 333148 | 301512 | 335215 | 462344 |
| Trial 3 | 9779 | 35282 | 30872 | 139987 | 206279 | 220897 | 328389 | 300488 | 461247 |
| Average | 14639 | 34597 | 39108 | 151766.76 | 224397 | 291874.9 | 321771 | 326741 | 463147 |
| Binding | 0.034285 | 0.08102 | 0.091588 | 0.355419 | 0.52551 | 0.683535 | 0.75355 | 0.76519 |  |
| **NBS** | **1.00E-09** | **5.00E-09** | **1.00E-08** | **5.00E-08** | **1.00E-07** | **5.00E-07** | **1.00E-06** | **5.00E-06** | **Probe** |
| Trial 1 | 19985 | 37885 | 34458 | 43528 | 51985 | 68555 | 55512 | 71445 | 465664 |
| Trial 2 | 17753 | 37521 | 32141 | 41152 | 47880 | 64118 | 52998 | 66529 | 463887 |
| Trial 3 | 17636 | 34577 | 34782 | 42211 | 52763 | 67915 | 53950 | 74516 | 460014 |
| Average | 18457 | 36661 | 33793 | 42296 | 50875 | 66862 | 54153 | 70830 | 463188 |
| Binding | 0.043123 | 0.08565 | 0.078952 | 0.098818 | 0.11886 | 0.156212 | 0.12652 | 0.16548 |  |

This table includes nitrocellulose filter binding data determining the relative binding of *At*MYB61 to the CASTing targets and to the mutated ACCTAC motifs in triplicate. The 60 bp DNA probes were present in excess amounts. The probe concentrations for each sequence was 1065nM and the total amount of DNA added to each reaction was 124.41ng. The protein concentrations are labelled in red and vary from 0M to 5.00E ^-09^ M. The cpm of each sample was measured by a liquid scintillation counter. If *At*MYB61 bound to a sequence then it would reach a binding max of ~0.75 binding. If *At*MYB61 did not bind to a sequence, then the binding would not increase with the increase in protein concentration.

**Binding** = Average binding cpm (DNA+protein) – Average background cpm (DNA alone passed through filter)

Average probe cpm (124.41ng of labelled sequence used in each reaction) – Average background cpm
